# Lifestyle factors and prostate-specific antigen (PSA) testing in UK Biobank: Implications for epidemiological research

**DOI:** 10.1016/j.canep.2016.09.010

**Published:** 2016-12

**Authors:** Thomas J. Littlejohns, Ruth C. Travis, Tim J. Key, Naomi E. Allen

**Affiliations:** a Clinical Trial Service Unit and Epidemiological Studies Unit, Nuffield Department of Population Health, University of Oxford, Oxford, OX3 7LF, UK; b Cancer Epidemiology Unit, Nuffield Department of Population Health, University of Oxford, Oxford, OX3 7LF, UK

**Keywords:** BMI, body mass index, CI, confidence interval, OR, odds ratio, PSA, prostate-specific antigen, Prostate-specific antigen, Epidemiologic bias, Epidemiologic methods, Life style, Diet, Prostate cancer

## Abstract

**Background:**

The central role of prostate-specific antigen (PSA) testing in the diagnosis of prostate cancer leads to the possibility that observational studies that report associations between risk factors and prostate cancer could be affected by detection bias. This study aims to investigate whether reported risk factors for prostate cancer are associated with PSA testing in a large middle-aged population-based cohort in the UK.

**Methods:**

The cross-sectional association between a wide range of sociodemographic, lifestyle, dietary and health characteristics with PSA testing was examined in 212,039 men aged 40–69 years in UK Biobank.

**Results:**

A total of 62,022 (29%) men reported they had ever had a PSA test. A wide range of factors was associated with a higher likelihood of PSA testing including age, height, education level, family history of prostate cancer, black ethnic origin, not being in paid/self-employment, living with a wife or partner, having had a vasectomy, being diagnosed with cancer or hypertension and having a high dietary intake of cereal, cooked and salad/raw vegetables, fresh fruit and tea. Conversely, socioeconomic deprivation, Asian ethnic origin, current smoking, low alcohol intake, high body-mass index, high coffee consumption and being diagnosed with diabetes, heart disease or stroke were associated with a lower likelihood of PSA testing.

**Conclusions:**

A variety of sociodemographic, lifestyle and health-related characteristics are associated with PSA testing, suggesting that observed associations of some of these traits with risk for prostate cancer in epidemiological studies may be, at least partially, due to detection bias.

## Highlights

• Prostate-specific antigen (PSA) testing is key in diagnosing prostate cancer.
• Risk factors for prostate cancer were related to the likelihood of PSA testing.
• There is potential for detection bias in epidemiological studies of prostate cancer.

## 1 Introduction

The only well-established lifestyle and demographic risk factors for prostate cancer are advanced age, being of black ethnic origin and having a family history of the disease. A wide range of other sociodemographic, behavioural, dietary and health-related characteristics have also been reported to be associated with increased prostate cancer risk [1], although these findings are less well established between studies and across populations. This inconsistency may, in part, be due to detection bias if these characteristics are also associated with the likelihood of having had a prostate-specific antigen (PSA) test, which is a key part of the diagnostic pathway for prostate cancer.

Enhanced detection through PSA testing largely explains the increased incidence of prostate cancer over the last 20 years in many countries [2], [3]. In the UK, although PSA testing is not currently recommended as a screening tool for prostate cancer [4], it is widely performed in primary care, either as a frontline test for men presenting with urinary tract/prostatic symptoms or as a free test for men aged ≥50 years at the request of the patient [5]. The aim of this study was to examine the associations between a wide range of sociodemographic, lifestyle and health-related characteristics and PSA testing in a large UK cohort without a routine screening programme, with a particular focus on established or possible risk factors for prostate cancer.

## 2 Material and methods

Participants were selected from UK Biobank, a large population-based cohort study that recruited 502,649 men and women aged between 40 and 69 years in 2006–2010 throughout the UK. UK Biobank received ethical approval from the National Health Service North West Centre for Research Ethics Committee (Ref: 11/NW/0382). The assessment visit comprised electronic signed consent, a touch-screen questionnaire, a brief computer-assisted interview, physical measures, and collection of blood, urine and saliva samples [6]. Of the 229,182 men recruited, data on PSA testing were obtained from 228,715 (99.8%) men who responded to the question ‘*Have you ever had a blood test for prostate cancer (prostate specific antigen or PSA test)?*’. Of these, 216,289 (94.6%) responded either yes or no; the remaining 12,426 participants who responded with ‘*Do not know*’ or ‘*Prefer not to answer*’ were excluded from the analysis. A further 4,250 men with prevalent prostate cancer (based on cancer registry data), benign prostatic hyperplasia or prostatitis (via self-report at recruitment) were excluded, resulting in a final sample size of 212,039 men.

Logistic regression models were used to investigate the association between baseline characteristics and ever having had a PSA test after adjustment for age, Townsend deprivation score and education. Models were performed with additional adjustment for region, family history of prostate cancer, ethnicity, employment, living with a wife or partner, smoking status, alcohol intake, body-mass index (BMI), standing height and private healthcare. A test for linear trend was obtained by entering the categorical variable into the model as a continuous variable; a test for difference in odds ratios between categories of the exposures of interest was obtained using the likelihood ratio test.

We also examined whether baseline characteristics of men who undergo PSA testing because of prostate symptoms differ from those of men who may request a PSA test as part of the National Health Service prostate cancer risk management programme available to men aged ≥50 years. Age at PSA testing was derived from the question ‘*How many years ago was your last test*? which was asked of men who responded that they had ever had a PSA test. For all characteristics, a likelihood ratio was used to test for heterogeneity between age groups (<50 vs. ≥50 years). For characteristics for which there was significant heterogeneity in the association with PSA-testing by age group, logistic regression models stratified by age group were performed. All *P-*values were two-sided with statistical significance set at <0.05. Statistical analyses were performed using Stata/SE version 13.1 (StataCorp LP, College Station, Texas).

## 3 Results

Of the 212,039 men included in the analyses, 62,022 (29%) reported ever having had a PSA test. A wide range of sociodemographic, lifestyle and health-related characteristics were significantly associated with PSA testing (Table 1). Age was most strongly associated with PSA testing (fully adjusted odds ratio [OR] for 65–70 years vs. 40–44 years = 18.8, 95% confidence interval [CI] 17.6–20.2). Men who had a father or brother with prostate cancer were twice as likely to have ever had a PSA test (OR = 1.92, 95% CI 1.86–2.00) compared to men with no family history; whilst men who had a father and a brother with prostate cancer were more than five times as likely (OR = 5.32, 95% CI 4.13–6.85, *p-*value for linear trend <0.001). Men who were more highly educated, were of black ethnic origin, not in paid/self-employment, living with a wife or partner, had private healthcare, were taller, had a vasectomy or who were diagnosed with (non-prostate) cancer or hypertension were also more likely to have ever had a PSA test. Conversely, men who lived in more socioeconomically deprived areas, were current smokers, had a lower alcohol intake, a higher BMI or who had been diagnosed with diabetes, heart disease or stroke were less likely to have had a PSA test. There was also regional variation, with PSA testing being least common in the East Midlands and North East England and most common in Wales and South West England. Mutual adjustment for these factors made little difference to the observed associations.

Several dietary characteristics were also associated with PSA testing (Table 2), with a higher intake of cereal, cooked vegetables, salad/raw vegetables, fresh fruit and tea consumption associated with a higher likelihood of PSA testing, whereas a higher coffee consumption was associated with a lower likelihood of PSA testing.

The associations of several sociodemographic and lifestyle factors with PSA testing differed by age at testing, with generally stronger associations found in men aged ≥50 years compared to men aged <50 years (Table 3).

## 4 Discussion

We examined the relationship between a wide range of putative risk factors for prostate cancer, including sociodemographic, lifestyle, dietary and health characteristics, and the likelihood of having had a PSA test in a population without a routine PSA-screening programme. These findings are important because of the central role of PSA testing in the diagnosis of prostate cancer, which leads to the possibility that associations reported between risk factors and prostate cancer in observational studies could be affected by detection bias (i.e. factors that are associated with men choosing to undergo PSA testing will, in turn, be associated with increased prostate cancer incidence).

In this large population-based cohort of middle-aged UK men, PSA testing was independently associated with age, a family history of prostate cancer, higher education, living with a wife or partner, private healthcare, height, having had a vasectomy, being diagnosed with cancer or hypertension and consuming a healthy diet. In contrast, socioeconomic deprivation, current smoking, having a lower alcohol intake, a higher BMI, a higher coffee consumption and being diagnosed with diabetes, heart disease or stroke were associated with less PSA testing. Our findings are consistent with a small number of studies based on general practitioner records conducted in the UK that found that men who are older [7], [8], [9], [10], less socioeconomically deprived [7], [8], [9], [10] and who live in southern UK regions [8] are more likely to undergo PSA testing, whereas men of Asian ethnic origin [7] are less likely to undergo PSA testing. We also found that men of black ethnic origin were more likely to have had a PSA test, independent of other factors such as family history and education status.

Many lifestyle characteristics reported here as being associated with PSA testing have also been associated with an increased risk of prostate cancer incidence, including family history [11], black ethnic origin [12] and height [13]. Conversely, factors associated with a lower rate of PSA testing have been associated with a lower risk of prostate cancer, such as obesity [14], diabetes [15], being of Asian ethnic origin [16] and smoking [17]. This suggests that the magnitude of associations observed with risk of total prostate cancer for established risk factors such as black ethnic origin and family history might be exaggerated in recent studies, whereas less well-established associations (such as those between dietary factors and prostate cancer risk), might be due to detection bias.

This study has several strengths. Due to the breadth and depth of information available in UK Biobank, we were able to explore a wide range of sociodemographic, lifestyle and health characteristics in relation to PSA testing, including characteristics previously identified as risk factors for prostate cancer. Our finding that socioeconomic deprivation, current smoking, higher BMI and a diagnosis of heart disease or stroke were more strongly associated with a lower likelihood of PSA testing in older men (who are more likely to choose to have a PSA test) compared with younger men (who are more likely to have a PSA test due to symptoms) [5], suggests that, among older men, the decision to request a PSA test is highly determined by lifestyle and sociodemographic factors in this population. This may have implications in interpreting the relationships between lifestyle factors and subsequent prostate cancer incidence.

However, this study also has several limitations. UK Biobank is a self-selected sample, with 29% of men reporting they had ever had a PSA test, which is substantially higher than a previous study in the UK general population that estimated the prevalence of PSA testing in asymptomatic men aged 45–89 years to be about 6% in the same time period [8]. Nonetheless, the findings from the current study do highlight the potential role of detection bias in epidemiological studies when investigating risk factors for prostate cancer in most Western populations where PSA testing is correlated with health-seeking behaviour. PSA testing was determined by self-report which may be subject to misclassification bias if, for example, men were unaware of having had a PSA test or had forgotten about it. Furthermore, the study did not collect information on the timing, frequency, reason or the outcome of each PSA test. Future linkage of UK Biobank data to primary care records will help us to address some of these limitations and to expand the analyses to the exploration of factors associated with PSA testing over time.

## 5 Conclusions

This population-based study shows the associations between a wide range of sociodemographic, lifestyle, dietary and health characteristics and the likelihood of PSA testing in a large UK cohort. These results indicate that it is important to consider the role of detection bias in epidemiological studies investigating risk factors for prostate cancer.

## Conflicts of interest

None.

## Author’s contributions

All authors were responsible for the conception and design of the study as well as the interpretation of the data. TJL analysed the data and drafted the manuscript. RCT, TJK and NEA revised the manuscript for important intellectual content. All authors gave final approval of the manuscript to be published.

## Funding

This work was supported by Cancer Research UK (grant number C8221/A19170). The funding source did not have any role in the design of the study; in the analysis and interpretation of the data; or in the preparation of the manuscript.

## Figures and Tables

**Table 1:** Logistic regression models investigating the association between baseline characteristics and having had a PSA test.

Characteristic	Ever had a PSA test, No. (%)		Model A^a^		Model B^b^	
	No	Yes	OR (95% CI)	p-value	OR (95% CI)	p-value
Age group (years)						
40–44^c^	21,858 (95.5)	1,043 (4.6)	1 (Reference)		1 (Reference)	
45–49	25,299 (91.4)	2,391 (8.6)	1.98 (1.84–2.13)		1.99 (1.85–2.15)	
50–54	25,293 (81.3)	5,814 (18.7)	4.83 (4.51–5.17)		4.87 (4.54–5.21)	
55–59	25,611 (68.6)	11,734 (31.4)	9.64 (9.03–10.3)		9.64 (9.01–10.3)	
60–64	29,718 (58.6)	21,026 (41.4)	15.2 (14.2–16.2)		14.8 (13.8–15.8)	
65–70^d^	22,238 (52.6)	20,014 (47.4)	20.0 (18.7–21.4)	<0.001^e^	18.8 (17.6–20.2)	<0.001^e^

Townsend deprivation score (quintiles)						
One (most affluent)	27,997 (66.1)	14,377 (33.9)	1 (Reference)		1 (Reference)	
Two	28,870 (68.2)	13,472 (31.8)	0.92 (0.89–0.95)		0.93 (0.90–0.96)	
Three	29,718 (70.2)	12,648 (29.9)	0.87 (0.84–0.90)		0.90 (0.87–0.92)	
Four	30,752 (72.7)	11,579 (27.4)	0.84 (0.82–0.87)		0.88 (0.85–0.90)	
Five (most deprived)	32,461 (76.7)	9,885 (23.3)	0.75 (0.72–0.77)	<0.001^e^	0.84 (0.81–0.87)	<0.001^e^

Education						
No qualifications	24,503 (68.9)	11,037 (31.1)	1 (Reference)		1 (Reference)	
CSE/O-Level/GCSE or equivalent	21,540 (75.2)	7,111 (24.1)	1.16 (1.12–1.21)		1.08 (1.04–1.13)	
AS/A-Level or equivalent	7,764 (72.6)	2,926 (27.4)	1.32 (1.25–1.39)		1.16 (1.10–1.23)	
Higher education or other professional qualification, or equivalent	93,415 (70.1)	39,858 (29.9)	1.42 (1.39–1.46)	<0.001^e^	1.27 (1.23–1.31)	<0.001^e^

Region						
London	18,858 (67.6)	9,031 (32.38)	1 (Reference)		1 (Reference)	
South-West	11,671 (65.4)	6,187 (34.7)	1.02 (0.97–1.06)		1.08 (1.03–1.13)	
South-East	12,491 (69.1)	5,595 (30.9)	0.79 (0.75–0.82)		0.86 (0.82–0.90)	
Wales	5,778 (65.0)	3,116 (35.0)	1.05 (1.00–1.11)		1.16 (1.10–1.23)	
West Midlands	14,404 (71.7)	5,674 (28.3)	0.74 (0.71–0.77)		0.79 (0.75–0.82)	
East Midlands	10,743 (74.8)	3,616 (25.2)	0.55 (0.53–0.58)		0.59 (0.56–0.63)	
Yorkshire & Humber	22,814 (72.6)	8,604 (27.4)	0.67 (0.64–0.69)		0.71 (0.68–0.74)	
North-West	24,415 (71.7)	9,647 (28.3)	0.72 (0.70–0.75)		0.78 (0.75–0.82)	
North-East	18,159 (74.0)	6,373 (26.0)	0.62 (0.60–0.65)		0.67 (0.64–0.70)	
Scotland	10,684 (71.9)	4,179 (28.1)	0.74 (0.70–0.77)	<0.001^f^	0.82 (0.78–0.86)	<0.001^f^

Population density						
Urban	128,703 (71.2)	52,093 (28.8)	1 (Reference)		1 (Reference)	
Rural	19,457 (67.4)	9,404 (32.6)	1.01 (0.98–1.04)	0.52	1.01 (0.98–1.04)	0.49

Family history of prostate cancer						
No	138,451 (71.9)	54,205 (28.1)	1 (Reference)		1 (Reference)	
Brother or father	9,114 (57.8)	6,665 (42.2)	1.96 (1.89–2.03)		1.92 (1.86–2.00)	
Brother and father	93 (27.4)	247 (72.7)	5.21 (4.06–6.69)	<0.001^e^	5.32 (4.13–6.85)	<0.001^e^

Ethnicity						
White	140,176 (70.2)	59,570 (29.8)	1 (Reference)		1 (Reference)	
Mixed background	805 (78.8)	217 (21.2)	1.11 (0.94–1.31)		1.07 (0.91–1.26)	
Black	2,433 (77.8)	695 (22.2)	1.36 (1.24–1.50)		1.29 (1.17–1.41)	
Asian	4,506 (82.9)	927 (17.1)	0.68 (0.63–0.73)		0.65 (0.60–0.71)	
Other	1,460 (80.7)	350 (19.3)	0.97 (0.85–1.10)	<0.0001^f^	0.95 (0.83–1.08)	<0.001^f^

Employment						
Paid/self-employment	100,556 (77.3)	29,459 (22.7)	1 (Reference)		1 (Reference)	
Not in paid/self-employment	48,040 (60.0)	32,043 (40.0)	1.07 (1.04–1.09)	<0.001	1.14 (1.11–1.16)	<0.001

Lives with a wife or partner						
No	26,801 (74.6)	9,140 (25.4)	1 (Reference)		1 (Reference)	
Yes	112,178 (69.1)	50,170 (30.9)	1.23 (1.20–1.27)	<0.001	1.21 (1.17–1.24)	<0.001

Smoking						
Never	75,246 (72.1)	29,084 (27.9)	1 (Reference)		1 (Reference)	
Former	53,248 (66.1)	27,351 (33.9)	1.01 (0.99–1.03)		1.00 (0.98–1.02)	
Current—Only occasionally	5,677 (76.2)	1,775 (23.8)	0.91 (0.85–0.96)		0.88 (0.83–0.94)	
Current—On all or most Days	15,298 (81.1)	3,567 (18.9)	0.65 (0.63–0.68)	<0.001^f^	0.67 (0.64–0.70)	<0.001^f^

Alcohol intake						
Never	9,813 (73.8)	3,481 (26.2)	0.95 (0.91–0.99)		1.00 (0.95–1.04)	
Special occasions only	11,304 (73.6)	4,064 (26.4)	0.92 (0.88–0.96)		0.94 (0.90–0.98)	
One to three times a month	14,038 (74.7)	4,750 (25.3)	0.95 (0.91–0.99)		0.96 (0.92–1.00)	
Once or twice a week	39,956 (72.7)	15,010 (27.3)	1 (Reference)		1 (Reference)	
Three or four times a week	38,966 (70.1)	16,596 (29.9)	1.03 (1.00–1.06)		1.01 (0.98–1.03)	
Daily or almost daily	35,775 (66.4)	18,086 (33.6)	1.06 (1.03–1.09)	<0.001^e^	1.03 (1.00–1.06)	<0.001^e^

BMI (kg/m^2^)						
<18.5	399 (80.6)	96 (19.4)	0.65 (0.51–0.82)		0.74 (0.59–0.94)	
≥18.5 ≤ 25	37,654 (71.2)	15,234 (28.8)	1 (Reference)		1 (Reference)	
≥25 ≤ 30	72,875 (69.8)	31,479 (30.2)	1.01 (0.99–1.04)		1.00 (0.98–1.03)	
≥30 ≤ 35	29,364 (71.4)	11,791 (28.7)	0.95 (0.92–0.98)		0.95 (0.92–0.98)	
≥35 ≤ 40	6,660 (73.0)	2,464 (27.0)	0.91 (0.87–0.96)		0.92 (0.87–0.97)	
≥40	2,078 (76.0)	655 (24.0)	0.86 (0.79–0.95)	<0.001^e^	0.87 (0.79–0.96)	<0.001^e^

Standing height (cm)						
<175	64,890 (70.4)	27,342 (29.6)	1 (Reference)		1 (Reference)	
≥175 ≤ 180	41,620 (70.4)	17,520 (29.6)	1.10 (1.07–1.13)		1.07 (1.04–1.10)	
≥180	42,714 (71.6)	16,921 (28.4)	1.20 (1.17–1.23)	<0.001^e^	1.14 (1.11–1.17)	<0.001^e^

Private healthcare						
No	36,665 (72.6)	13,825 (27.4)	1 (Reference)		1 (Reference)	
Yes	12,457 (60.1)	8,289 (40.0)	1.86 (1.80–1.94)	<0.001	1.78 (1.71–1.85)	<0.001

Vasectomy (self-report)						
No	142,444 (70.8)	58,675 (29.2)	1 (Reference)		1 (Reference)	
Yes	7,573 (69.4)	3,347 (30.7)	1.10 (1.05–1.15)	<0.001	1.07 (1.02–1.12)	0.006

Cancer (cancer registry)^g^						
No	143,158 (71.43)	57,270 (28.57)	1 (Reference)		1 (Reference)	
Yes	6,859 (59.07)	4,752 (40.93)	1.22 (1.17–1.27)	<0.001	1.22 (1.17–1.27)	<0.001

Diabetes (self-report)						
No	140,235 (70.9)	57,487 (29.1)	1 (Reference)		1 (Reference)	
Yes	9,782 (68.3)	4,535 (31.7)	0.86 (0.83–0.89)	<0.001	0.90 (0.87–0.94)	<0.001

Heart disease (self-report)						
No	138,464 (71.3)	55,727 (28.7)	1 (Reference)		1 (Reference)	
Yes	11,553 (64.7)	6,295 (35.3)	0.91 (0.88–0.94)	<0.001	0.95 (0.92–0.98)	0.003

Hypertension (self-report)						
No	109,391 (73.3)	39,833 (26.7)	1 (Reference)		1 (Reference)	
Yes	40,626 (64.7)	22,189 (35.3)	1.09 (1.06–1.11)	<0.001	1.11 (1.09–1.14)	<0.001

Stroke (self-report)						
No	146,942 (70.9)	60,421 (29.1)	1 (Reference)		1 (Reference)	
Yes	3,075 (65.8)	1,601 (34.2)	0.90 (0.84–0.96)	0.001	0.93 (0.87–0.99)	0.03

Abbreviations: BMI, body mass index; CI, confidence interval; OR, odds ratio; PSA, prostate-specific antigen.

a Adjusted for age, Townsend deprivation score and education.

b Adjusted for age, Townsend deprivation score, education, region, family history of prostate cancer, ethnicity, employment, married/partner, smoking, alcohol intake, BMI, standing height and private healthcare.

c Includes 4 participants who were <40 at baseline (minimum age = 38).

d Includes 1099 participants who >69 at baseline (maximum age = 73).

e Test for linear trend.

f Test for heterogeneity.

g Prostate cancer cases excluded.

**Table 2:** Logistic regression models investigating the association between dietary characteristics and having had a PSA test.

Characteristic	Ever had a PSA test, No. (%)		Model A^a^		Model B^b^	
	No	Yes	OR (95% CI)	p-value	OR (95% CI)	p-value
Cereal intake (bowls/week)						
<1	28,776 (75.8)	9,173 (24.2)	1 (Reference)		1 (Reference)	
1–3	29,348 (74.4)	10,083 (25.6)	1.12 (1.09–1.16)		1.11 (1.08–1.15)	
4–6	39,569 (71.5)	15,775 (28.5)	1.22 (1.19–1.26)		1.18 (1.14–1.22)	
≥7	51,509 (65.8)	26,810 (34.2)	1.30 (1.26–1.34)	<0.001^c^	1.26 (1.22–1.30)	<0.001^c^

Cooked vegetable intake (servings/day)						
<1	9,964 (80.0)	2,490 (20.0)	1 (Reference)		1 (Reference)	
1	22,982 (75.4)	7,493 (24.6)	1.14 (1.08–1.21)		1.07 (1.01–1.13)	
2	46,753 (70.6)	19,469 (29.4)	1.22 (1.16–1.28)		1.11 (1.05–1.17)	
≥3	67,564 (68.0)	31,814 (32.0)	1.26 (1.20–1.32)	<0.001^c^	1.14 (1.09–1.20)	<0.001^c^

Salad/raw vegetable intake (servings/day)						
<1	29,130 (73.5)	10,488 (26.5)	1 (Reference)		1 (Reference)	
1	47,673 (70.9)	19,579 (29.1)	1.22 (1.09–1.16)		1.07 (1.04–1.10)	
2	33,370 (69.5)	14,632 (30.5)	1.16 (1.12–1.20)		1.10 (1.06–1.13)	
≥3	37,197 (69.3)	16,509 (30.7)	1.17 (1.13–1.21)	<0.001^c^	1.11 (1.07–1.14)	<0.001^c^

Fresh fruit intake (servings/day)						
<1	20,181 (77.8)	5,748 (22.2)	1 (Reference)		1 (Reference)	
1	45,844 (71.6)	18,157 (28.4)	1.20 (1.15–1.24)		1.14 (1.09–1.18)	
2	39,460 (69.3)	17,482 (30.7)	1.29 (1.24–1.34)		1.21 (1.17–1.26)	
≥3	43,648 (68.1)	20,415 (31.87)	1.37 (1.32–1.42)	<0.001^c^	1.30 (1.25–1.35)	<0.001^c^

Tea consumption (cups/day)						
<1	27,722 (75.3)	9,080 (24.7)	1 (Reference)		1 (Reference)	
1–2	35,466 (70.8)	14,655 (29.2)	1.17 (1.13–1.21)		1.14 (1.10–1.17)	
3–4	41,345 (68.3)	19,175 (31.7)	1.19 (1.15–1.23)		1.16 (1.12–1.20)	
≥5	45,126 (70.3)	19,024 (29.7)	1.12 (1.09–1.16)	<0.001^c^	1.13 (1.09–1.16)	<0.001^c^

Coffee consumption (cups/day)						
<1	41,932 (72.4)	15,984 (27.6)	1 (Reference)		1 (Reference)	
1–2	54,126 (67.8)	25,656 (32.2)	1.09 (1.06–1.12)		1.05 (1.02–1.07)	
≥3	53,509 (72.5)	20,275 (27.5)	0.94 (0.92–0.97)	<0.001^c^	0.93 (0.91–0.96)	<0.001^c^

Abbreviations: CI, confidence interval; OR, odds ratio; PSA, prostate-specific antigen.

a Adjusted for age, Townsend deprivation score and education.

b Adjusted for age, Townsend deprivation score, education, region, family history of prostate cancer, ethnicity, employment, married/partner, smoking, alcohol intake, BMI, standing height and private healthcare.

c Test for linear trend.

**Table 3:** The association between characteristics and having had a PSA test stratified by age at testing.^a^^,^^b^

Characteristic	<50 years old at time of PSA test (n = 7106)		≥50 years old at time of PSA test (*n* = 53,112)		p-value for difference by age at PSA test^c^
	OR (95% CI)	p-value	OR (95% CI)	p-value	
Townsend deprivation score (quintiles)					
One (most affluent)	1 (Reference)		1 (Reference)		
Two	0.89 (0.83–0.95)		0.94 (0.90–0.97)		
Three	0.89 (0.83–0.95)		0.90 (0.87–0.93)		
Four	0.90 (0.84–0.97)		0.87 (0.84–0.90)		
Five (most deprived)	0.93 (0.86–1.00)	0.08^d^	0.83 (0.80–0.86)	<0.001^d^	<0.001

Region					
London	1 (Reference)		1 (Reference)		
South-West	0.87 (0.79–0.96)		1.16 (1.10–1.22)		
South-East	0.84 (0.75–0.93)		0.88 (0.83–0.93)		
Wales	0.98 (0.87–1.11)		1.25 (1.17–1.33)		
West Midlands	0.81 (0.74–0.89)		0.80 (0.76–0.84)		
East Midlands	0.57 (0.51–0.64)		0.61 (0.58–0.65)		
Yorkshire & Humber	0.70 (0.64–0.76)		0.73 (0.69–0.76)		
North-West	0.74 (0.68–0.80)		0.80 (0.77–0.84)		
North-East	0.68 (0.62–0.75)		0.68 (0.65–0.72)		
Scotland	0.71 (0.64–0.79)	<0.001^e^	0.86 (0.81–0.91)	<0.001^e^	<0.001

Family history of prostate cancer					
No	1 (Reference)		1 (Reference)		
Brother or father	2.35 (2.21–2.51)		1.76 (1.69–1.83)		
Brother and father	4.39 (2.64–7.31)	<0.001^d^	5.03 (3.87–6.54)	<0.001^d^	<0.001

Smoking					
Never	1 (Reference)		1 (Reference)		
Former	1.03 (0.98–1.08)		0.99 (0.97–1.01)		
Current—Only occasionally	0.99 (0.89–1.11)		0.84 (0.79–0.90)		
Current—On all or most days	0.78 (0.72–0.85)	<0.001^e^	0.64 (0.61–0.67)	<0.001^e^	<0.001

Alcohol intake					
Never	1.07 (0.97–1.18)		0.98 (0.94–1.04)		
Special occasions only	1.03 (0.94–1.13)		0.92 (0.88–0.97)		
One to three times a month	0.96 (0.88–1.04)		0.96 (0.91–1.00)		
Once or twice a week	1 (Reference)		1 (Reference)		
Three or four times a week	0.96 (0.91–1.02)		1.01 (0.98–1.04)		
Daily or almost daily	0.93 (0.88–1.00)	0.004^d^	1.03 (1.00–1.06)	<0.001^d^	0.02

BMI (kg/m^2^)					
<18.5	0.85 (0.53–1.36)		0.70 (0.54–0.91)		
≥18.5 ≤ 25	1 (Reference)		1 (Reference)		
≥25 ≤ 30	0.97 (0.92–1.02)		1.00 (0.97–1.03)		
≥30 ≤ 35	0.94 (0.88–1.01)		0.94 (0.91–0.97)		
≥35 ≤ 40	0.98 (0.87–1.09)		0.91 (0.86–0.96)		
≥40	1.08 (0.91–1.30)	0.44^d^	0.84 (0.75–0.93)	<0.001^d^	0.001

Heart disease (self-report)					
No	1 (Reference)		1 (Reference)		
Yes	1.16 (1.06–1.27)	0.001	0.94 (0.91–0.97)	0.001	0.01

Stroke (self-report)					
No	1 (Reference)		1 (Reference)		
Yes	1.17 (0.99–1.38)	0.06	0.92 (0.86–0.98)	0.01	0.04

Fresh fruit intake (servings/day)					
<1	1 (Reference)		1 (Reference)		
1	1.06 (0.98–1.14)		1.15 (1.10–1.19)		
2	1.06 (0.98–1.14)		1.24 (1.19–1.29)		
≥3	1.18 (1.10–1.27)	<0.001^d^	1.31 (1.26–1.36)	<0.001^d^	0.04

Abbreviations: BMI, body mass index; CI, confidence interval; OR, odds ratio; PSA, prostate-specific antigen.

a Adjusted for age, Townsend deprivation score, education, region, family history of prostate cancer, ethnicity, employment, married/partner, smoking, alcohol intake, BMI, standing height and private healthcare.

b Includes only those characteristics that showed statistically significant heterogeneity by age group.

c Heterogeneity in odds ratios between subgroups defined by age at PSA testing adjusted for age, Townsend deprivation score, education, family history of prostate cancer, ethnicity, employment, smoking, BMI and longstanding illness.

d Test for linear trend.

e Test for difference.
